# A173 WHAT HAS CHANGED IN THE DIAGNOSIS OF CELIAC DISEASE IN CANADIANS OVER THE PAST 25 YEARS? RESULTS FROM THE STATE OF CELIAC IN CANADA SURVEY 2022

**DOI:** 10.1093/jcag/gwad061.173

**Published:** 2024-02-14

**Authors:** J A King, C McAulay, M Secord, M Pinto-Sanchez, J Turner, D Gidrewicz, S Case, D Duerksen

**Affiliations:** University of Calgary, Calgary, AB, Canada; Canadian Celiac Association, Mississauga, ON, Canada; Canadian Celiac Association, Mississauga, ON, Canada; McMaster University, Hamilton, ON, Canada; University of Alberta, Edmonton, AB, Canada; University of Calgary, Calgary, AB, Canada; Canadian Celiac Association, Mississauga, ON, Canada; University of Manitoba, Winnipeg, MB, Canada

## Abstract

**Background:**

Prior surveys of Canadians with celiac disease (CD) have demonstrated several challenges on the path to diagnosis. It has been over a decade since findings from the most recent survey were published.

**Aims:**

To compare the experiences of Canadian individuals prior to CD diagnosis across time.

**Methods:**

The State of Celiac Disease in Canada Survey is an online, nationwide survey distributed by Celiac Canada in December 2022. Over 75 questions were included, covering diagnostic pathways, presenting symptoms, and involvement of health care professionals (HCPs). Analysis was restricted to those who responded to having CD, dermatitis herpetiformis, or gluten ataxia, as well as provided the time since their diagnosis.

**Results:**

A total of 6052 participants were included: 1210 (20.0%) were diagnosed in the last two years, 1113 (18.4%) two to five years ago, 1136 (18.8%) five to 10 years ago, 1040 (17.2%) 10 to 15 years ago, 960 (15.9%) 15 to 25 years ago, and 593 (9.8%) more than 25 years ago. Diarrhea was reported in over 70% of respondents diagnosed over 25 years ago, compared to approximately 50% diagnosed in the last five years (Figure 1A). Conversely, 31% of individuals diagnosed over 25 years ago experienced brain fog compared to 57% of those diagnosed in the last two years. Approximately 25% of respondents diagnosed at least 15 years ago experienced symptoms for more than 20 years prior, compared to 15% of those diagnosed in the last two years (Table 1). There were no clear differences in the proportion of respondents who saw a family physician before diagnosis, but there was a drop in those consulting a gastroenterologist (60% diagnosed more than 25 years ago versus 43% diagnosed in the last five years, Figure 1B).

**Conclusions:**

Over time, there has been a shift in the clinical presentation of celiac disease, which may reflect improved detection of cases without gastrointestinal symptoms. Delays in diagnosis remain a concern but have also improved over time. The phenomenon of fewer patients consulting with gastroenterologists requires further investigation to understand the cause and impact on accurate diagnosis.

Delays in diagnosis across time since diagnosis

Note: 496 respondents did not report time from symptoms to diagnosis

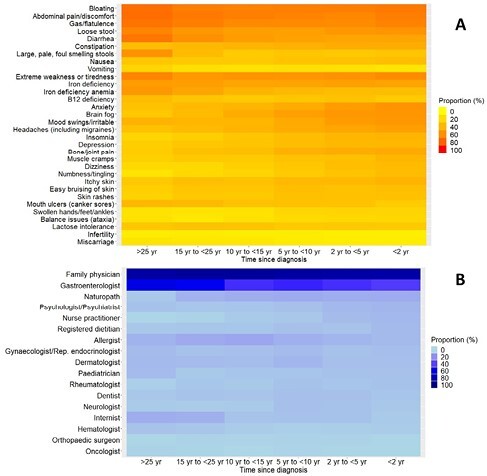

Symptoms experienced (A) and healthcare professionals consulted (B) prior to diagnosis

**Funding Agencies:**

Celiac Canada (FKA Canadian Celiac Association)

**Table:** 

Time since diagnosis from date of survey	Time from symptoms to diagnosis						
	No symptoms	ampersand:003C6 months	6 months to ampersand:003C2 years	2 to ampersand:003C5 years	5 to ampersand:003C10 years	10 to ampersand:003C20 years	ampersand:003E20 years
ampersand:003C2 years	64 (5.9%)	78 (7.2%)	229 (21.1%)	219 (20.2%)	181 (16.7%)	151 (13.9%)	164 (15.1%)
2 to ampersand:003C5 years	83 (8.1%)	64 (6.2%)	181 (17.6%)	188 (18.2%)	187 (18.1%)	132 (12.8%)	196 (19.0%)
5 to ampersand:003C10 years	84 (8.0%)	46 (4.4%)	195 (18.6%)	170 (16.2%)	157 (15.0%)	158 (15.0%)	240 (22.9%)
10 to ampersand:003C15 years	56 (5.8%)	56 (5.8%)	163 (17.0%)	158 (16.5%)	145 (15.1%)	154 (16.0%)	228 (23.8%)
15 to ampersand:003C25 years	31 (3.5%)	38 (4.3%)	146 (16.4%)	149 (16.7%)	138 (15.5%)	157 (17.6%)	233 (26.1%)
ampersand:003E25 years	7 (1.3%)	53 (9.9%)	110 (20.5%)	80 (14.9%)	71 (13.2%)	86 (16.0%)	130 (24.2%)

